# Salivary Biomarkers as a Predictive Factor in Anxiety, Depression, and Stress

**DOI:** 10.3390/cimb47070488

**Published:** 2025-06-26

**Authors:** Dana Gabriela Budala, Ionut Luchian, Dragos Ioan Virvescu, Teona Tudorici, Vlad Constantin, Zinovia Surlari, Oana Butnaru, Dan Nicolae Bosinceanu, Cosmin Bida, Monica Hancianu

**Affiliations:** 1 Department of Dentures, Faculty of Dental Medicine, “Grigore T. Popa” University of Medicine and Pharmacy, 700115 Iasi, Romania; 2 Department of Periodontology, Faculty of Dental Medicine, “Grigore T. Popa” University of Medicine and Pharmacy, 700115 Iasi, Romania; 3 Department of Dental Materials, Faculty of Dental Medicine, “Grigore T. Popa” University of Medicine and Pharmacy, 700115 Iasi, Romania; 4 Department of Fixed Prosthodontics, Faculty of Dental Medicine, “Grigore T. Popa” University of Medicine and Pharmacy, 700115 Iasi, Romania; 5 Department of Biophysics, Faculty of Dental Medicine, “Grigore T. Popa” University of Medicine and Pharmacy, 700115 Iasi, Romania; 6 Department of Pharmacognosy, Faculty of Pharmacy, “Grigore T. Popa” University of Medicine and Pharmacy, 16 University Street, 700115 Iasi, Romania

**Keywords:** saliva, biomarkers, stress, anxiety, depression, cortisol, alpha-amylase, BDNF, miRNAs, C-reactive protein

## Abstract

Anxiety and depression are highly prevalent mental health disorders often associated with dysregulation of neuroendocrine and immune systems, particularly the hypothalamic–pituitary–adrenal (HPA) axis and the sympathetic–adrenal–medullary (SAM) system. Recent research highlights the potential of salivary biomarkers to serve as non-invasive indicators for psychological distress. This narrative review synthesizes current evidence on key salivary biomarkers, cortisol, alpha-amylase (sAA), secretory immunoglobulin A (sIgA), chromogranin A (CgA), interleukin-6 (IL-6), tumor necrosis factor-alpha (TNF-α), C-reactive protein (CRP), brain-derived neurotrophic factor (BDNF), and salivary microRNAs (miRNAs), in relation to anxiety, depression, and stress. A comprehensive literature search (2010–2025) was conducted using multiple databases and relevant MeSH terms. The review reveals consistent associations between these salivary analytes and stress-related disorders, reflecting changes in neuroendocrine activity, immune response, and neuroplasticity. Cortisol and sAA mirror acute stress reactivity, while cytokines and CRP indicate chronic inflammation. BDNF and miRNAs provide insight into neuroplastic dysfunction and gene regulation. Despite promising results, limitations such as variability in sampling methods and biomarker specificity remain. In conclusion, salivary biomarkers offer a promising avenue for early detection, monitoring, and personalization of treatment in mood and anxiety disorders. Conclusions: Cortisol and alpha-amylase serve as the principal markers of acute stress response, whereas cytokines such as IL-6 and TNF-α, together with CRP, indicate chronic inflammation associated with extended emotional distress.

## 1. Introduction

The most common mental health conditions that cause impairment and early death are major depressive disorder (MDD) and generalized anxiety disorder (GAD) [1]. According to estimates, 4.4% of the world’s population, or more than 300 million individuals, suffer from serious depressive disorders [2]. Anxiety disorders affect a similarly large segment of the global population as depression, with the two conditions frequently coexisting and compounding each other’s impact. According to estimates from the World Health Organization (WHO), depression represents the leading cause of disability worldwide, accounting for approximately 7.5% of all years lived with disability (YLD) in 2015 [3].

In comparison, anxiety disorders rank as the sixth most significant contributor, responsible for around 3.4% of global YLD [4]. These figures not only underscore the enormous burden these mental health disorders place on individuals and health care systems but also highlight the urgent need for early detection and more accessible, non-invasive tools for monitoring psychological distress [5]. Given their high prevalence and frequent comorbidity, depression and anxiety are increasingly being studied together, particularly in relation to physiological biomarkers that might support better screening, diagnosis, and treatment personalization [2].

From a neurobiological perspective, these disorders are strongly associated with dysregulation of the hypothalamic–pituitary–adrenal (HPA) axis, overactivation of the sympathetic nervous system, and chronic low-grade inflammation [6].

These interconnected systems modulate both psychological and physiological responses to stress, and their disruption has been implicated in the pathophysiology of mood and anxiety disorders. Alterations in neuroendocrine and immune function can manifest as measurable biological changes, providing a rationale for exploring objective biomarkers in the context of mental health [7].

In this context, saliva has emerged as a promising and non-invasive diagnostic medium [8]. Rich in biomarkers such as cortisol, alpha-amylase, immunoglobulins, and cytokines, saliva reflects both neuroendocrine and immunological activity. Its ease of collection, low cost, and stress-free nature make it particularly attractive for screening and monitoring psychological states in clinical and research settings [9].

This review aims to synthesize the current scientific literature on salivary biomarkers associated with anxiety, depression, and stress, focusing on the underlying biological and molecular mechanisms, current limitations in their clinical applicability, and outlining future directions for research and clinical implementation.

## 2. Literature Review

Salivary biomarkers as indications of psychological discomfort, such as stress, depression, and anxiety, have been the subject of an increasing amount of research in the last 20 years [10]. A wide range of analytes have been investigated in these studies, from long-standing ones like cortisol and alpha-amylase to newer ones like cytokines, immunoglobulins, and microRNAs.

Research on salivary biomarkers as possible indications of psychological discomfort has expanded in recent years, driven by a rising interest in the biological bases of mental health [11]. The dynamics of anxiety, sadness, and stress may frequently be reflected by changes in salivary composition, which include intricate interactions between the neuroendocrine, immunological, and autonomic systems [12].

Saliva offers a non-invasive, easily obtainable fluid that reflects both acute and chronic physiological responses to psychological challenges [13]. This has made it a particularly attractive target in studies aiming to identify markers that may assist in the detection, monitoring, or even prediction of emotional and cognitive disturbances [14].

This review was conducted using a narrative synthesis approach aimed at identifying and summarizing current evidence on the role of salivary biomarkers in anxiety, depression, and stress. The methodology was designed to capture both well-established and emerging literature relevant to the physiological mechanisms, diagnostic potential, and limitations of salivary analytes in the context of psychological distress.

A comprehensive literature search was performed using the following electronic databases: PubMed, Scopus, Web of Science, and ScienceDirect. The search included articles published from 2010 to 2025. The following keywords and MeSH terms were used in various combinations: “saliva”, “salivary biomarkers”, “cortisol”, “alpha amylase”, “cytokines”, “depression”, “anxiety”, “psychological stress”, “mental health”, “HPA axis”, “inflammation”, and “non-invasive diagnostics”. Boolean operators (AND, OR) were applied to refine the search and ensure the inclusion of relevant studies.

✓ Study Selection and Data Extraction.

After the removal of duplicates, titles and abstracts were screened for relevance. Full-text articles were reviewed to confirm eligibility. Data was extracted on study design, population, salivary biomarkers measured, collection methods, and key findings. Particular attention was paid to biological mechanisms, clinical associations, and study limitations.

✓ Data Synthesis.

Findings were synthesized narratively and grouped according to biomarker type and physiological relevance. Where available, the molecular pathways and proposed mechanisms linking each biomarker to psychological disorders were also examined.

✓ Inclusion criteria were original articles and reviews published in English; studies involving human participants; articles reporting salivary biomarkers in relation to anxiety, depression, or psychological stress; and peer-reviewed journal publications.
✓ Exclusion criteria were animal studies, studies without access to the full text, case reports, editorials, and conference abstracts without supporting data.

Given the increasing body of evidence supporting the relationship between salivary composition and psychological states, several biomarkers have emerged as particularly relevant due to their consistent presence and physiological significance.

While this review is descriptive in nature, particular effort was made to ensure that the selection of studies followed a clear and structured rationale. After initial database screening, only studies that reported original data or systematic insights into salivary biomarkers associated with anxiety, depression, or stress were retained. Priority was given to research with clearly defined populations, standardized saliva collection protocols (e.g., time of day, stimulated vs. unstimulated flow), and validated biomarker assays.

Furthermore, to ensure thematic coherence, preference was given to studies that linked biomarker levels with established pathophysiological pathways, such as the HPA axis, the autonomic nervous system, or immuno-inflammatory cascades. Both clinical and experimental studies were included, provided they offered mechanistic insight or contributed to biomarker validation.

Review articles were included only if they synthesized data from multiple original studies and provided relevant context or interpretative frameworks. In contrast, case reports, letters, and editorial comments were excluded due to limited generalizability.

### 2.1. Cortisol

Cortisol, also known as 17-hydroxy-corticosterone, is a steroid hormone produced by the adrenal cortex [15]. Receptors for this hormone are present in a wide variety of cell types, making it an important regulator and maintainer of general health. It has an effect on many different systems, including the cardiovascular, neurological, metabolic, musculoskeletal, respiratory, and reproductive systems [16,17].

**Figure 1:**
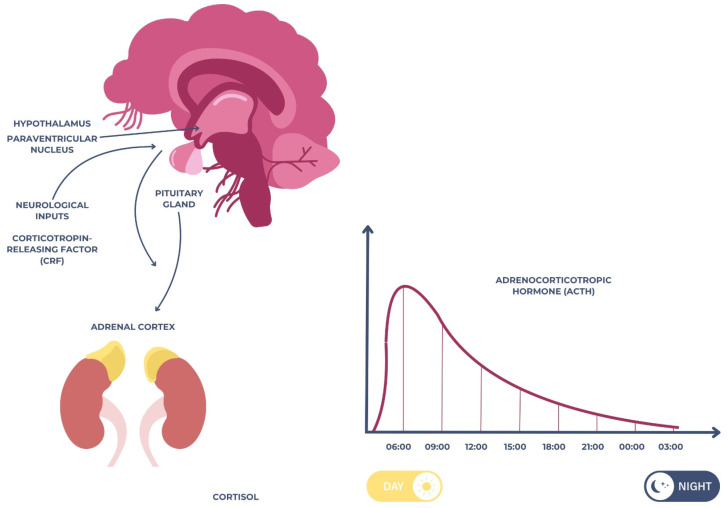
Schematic representation of the hypothalamic–pituitary–adrenal (HPA) axis.

Cortisol is one of the most extensively studied salivary biomarkers in relation to psychological stress, anxiety, and depression [18]. The stress response regulation system known as the hypothalamic–pituitary–adrenal (HPA) axis secretes the hormone cortisol. The hypothalamus, which receives neuronal impulses mostly from the paraventricular nucleus (PVN) and the suprachiasmatic nucleus, is the starting point of this circuit [19]. The anterior pituitary is stimulated to secrete adrenocorticotropic hormone (ACTH) when the PVN produces corticotropin-releasing factor (CRF) in response to stress-related stimuli [20]. The adrenal cortex is stimulated to produce more cortisol when ACTH enters the bloodstream. In healthy humans, cortisol levels rise in the first half an hour or so after waking up, fall throughout the day, and reach the lowest level during the night as part of a circadian cycle [21,22], as illustrated in Figure 1 below.

As the end-product of the hypothalamic–pituitary–adrenal (HPA) axis, cortisol plays a central role in the body’s response to stress. Under conditions of acute or chronic psychological pressure, the HPA axis is activated, resulting in increased secretion of cortisol into the bloodstream and its subsequent diffusion into saliva [23].

Numerous clinical studies have reported elevated salivary cortisol levels in individuals experiencing chronic stress and major depressive disorder (MDD) [24]. Salivary cortisol levels were found to be much higher in children who had tooth extractions, suggesting that they were under a great deal of stress [25]. In addition, cognitive–behavioral approaches and audiovisual diversions are known to alleviate dental anxiety and reduce cortisol levels, which in turn improves the dental experience for young patients. Better health outcomes and patient participation can be achieved by stress management in pediatric dental care, as highlighted by these data [26].

Elevated morning cortisol or a flattened diurnal cortisol slope has been associated with HPA axis dysregulation, which is frequently observed in patients with long-term psychological distress [27]. Moreover, altered cortisol patterns have been linked not only to symptom severity in depression and anxiety, but also to cognitive dysfunction, fatigue, and sleep disturbances. Deficits in memory and executive function are the most common forms of cognitive impairment in depressed people. In addition, both healthy controls and depressive patients have shown a correlation between elevated cortisol levels and poorer cognitive functioning [28,29].

However, interpretation of cortisol levels is complicated by its pronounced diurnal variation. Salivary cortisol follows a circadian rhythm, typically peaking within 30 min of awakening (the cortisol awakening response—CAR) and gradually declining throughout the day. Despite the fact that the CAR is linked to both past events and future stress, the exact neurobiological processes that cause it to form remain unclear [30].

This biological rhythm can be influenced by numerous factors, including age, sex, medication, sleep patterns, and comorbid medical conditions [31,32]. Therefore, timing and standardization of sample collection are critical for the reliability and reproducibility of findings [33].

In the 1980s, many research groups attempted to prove the clinical usefulness of the dexamethasone suppression test by examining its ability to reduce salivary cortisol and, by extension, depressed symptoms [34]. This sparked interest in studying the correlation between salivary cortisol levels and depression. In a study including 30 depressed inpatients ranging in age from 7 to 16, Foreman and Goodyer focused on juvenile depression and compared salivary hypercortisolism. There was a link between cortisol levels in saliva and depressive symptoms [35].

While elevated cortisol is often associated with stress and depressive symptoms, some studies have reported blunted or paradoxical responses, particularly in individuals with prolonged stress exposure or treatment-resistant depression [36]. These inconsistencies may reflect adaptive downregulation of the HPA axis over time, underscoring the importance of context when interpreting salivary cortisol data.

Despite its value, salivary cortisol is not without limitations [37]. Circadian variability, individual baseline differences, and the influence of comorbid conditions can complicate interpretation [38]. Furthermore, cortisol alone may not sufficiently differentiate between types of psychological distress. As such, it is increasingly recommended that cortisol be assessed in conjunction with additional biomarkers, such as alpha-amylase or pro-inflammatory cytokines, to improve diagnostic specificity and predictive power [39].

### 2.2. Salivary Alpha-Amylase (sAA)

Salivary alpha-amylase (sAA) is an enzyme secreted primarily by the salivary glands under sympathetic nervous system activation [40]. Unlike cortisol, which reflects hypothalamic–pituitary–adrenal (HPA) axis activity, sAA is considered a surrogate marker of sympathetic–adrenal–medullary (SAM) system function, offering complementary insights into physiological responses to stress [40]. In response to acute psychological or physical stressors, the central nervous system rapidly activates the sympathetic–adrenal–medullary (SAM) system [41]. This begins with the perception of a threat or stressor, which is processed by the amygdala and hypothalamus, particularly the paraventricular nucleus (PVN). The hypothalamus then triggers the sympathetic nervous system (SNS) through descending neuronal pathways that stimulate preganglionic sympathetic neurons located in the spinal cord [41].

These neurons synapse with postganglionic sympathetic fibers, which innervate the salivary glands, especially the parotid and submandibular glands. Upon activation, noradrenaline (norepinephrine) is released at the neuroeffector junctions. This catecholamine binds to β-adrenergic receptors (mainly β1 and β2) on acinar cells of the salivary glands [42].

**Figure 2:**
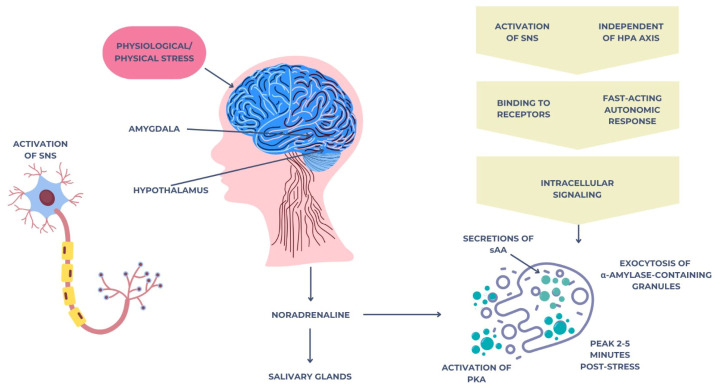
Neuroendocrine mechanism of salivary alpha-amylase secretion triggered by acute stress via the SAM pathway.

The binding of noradrenaline to these receptors activates adenylate cyclase, increasing intracellular cyclic adenosine monophosphate (cAMP) levels [43]. Elevated cAMP leads to the activation of protein kinase A (PKA), which in turn phosphorylates proteins involved in the exocytosis of alpha-amylase-containing secretory granules. As a result, large amounts of alpha-amylase are secreted into the saliva within 2–5 min after stress exposure [44]. This entire response is independent of the hypothalamic–pituitary–adrenal (HPA) axis and represents a fast-acting autonomic mechanism that reflects the immediate mobilization of the body’s resources to face acute challenges. The mechanism is illustrated in Figure 2 below.

Researchers have shown that people with post-traumatic stress disorder (PTSD), GAD, and MDD all have altered autonomic tone, and salivary alpha-amylase (sAA) may be a biomarker of this deregulation [45]. According to research, those who have more intense anxiety symptoms frequently have higher levels of sympathetic activation at baseline, which implies that they are chronically in a state of overactivity [46].

Individuals with certain psychopathologies may exhibit abnormalities in autonomic sensitivity or maladaptive reactivity in experimental contexts, such as the Trier Social Stress Test, which involves exposure to acute psychosocial stressors [47]. This contradictory reaction suggests that sAA has both trait-like features, suggesting a chronic autonomic imbalance, and state-like traits, reflecting the moment-to-moment changes in emotional and physiological responsiveness.

These results provide credence to the expanding body of evidence suggesting sAA is a flexible and adaptable biomarker that can shed light on the acute and chronic aspects of stress-related mental health disorders [48].

Salivary alpha-amylase (sAA) presents multiple clinical and translational applications in the field of mental health, owing to its rapid response to stress and the ease of non-invasive sample collection. It holds promise as a biomarker for the early identification of burnout and stress-related conditions in professional settings, supporting workplace health monitoring efforts [49].

The development of machine learning algorithms that can analyze multidimensional salivary biomarker profiles to predict individual vulnerability and treatment outcomes in anxiety, depression, and related conditions, as well as the investigation of genetic polymorphisms that impact sAA expression and its interaction with pro-inflammatory cytokines in chronic stress and mood disorders, are all areas that require further investigation [50].

### 2.3. Salivary Immunoglobulin A (sIgA)

Saliva and other mucosal secretions are primarily defended against pathogens by secretory immunoglobulin A (sIgA), the most abundant antibody in the body [51]. The lamina propria of salivary glands is home to plasma cells that synthesize sIgA. This protein then acquires a secretory component that makes it more stable in the harsh mucosal environment before being transferred across epithelial cells via the polymeric immunoglobulin receptor (pIgR) [52]. Its primary immunological role is to maintain mucosal homeostasis by neutralizing pathogens, preventing microbial adhesion, and modulating local immune responses without triggering inflammation [53].

Psychoneuroimmunology suggests that sIgA levels are responsive to emotional and mental states, especially stress (both short-term and long-term) [54]. In response to sympathetic stimulation, sIgA levels may temporarily rise during acute stress [55]. However, sustained exposure to glucocorticoids and dysregulation of autonomic input to the salivary glands have consistently been linked to suppressed sIgA secretion during chronic or long-term stress [56]. A decrease in sIgA levels weakens mucosal immunity, which in turn makes people more prone to infections and inflammatory diseases [57].

Evidence of decreased mucosal immune defense associated with neuroendocrine dysregulation has been found in people with anxiety and depression, who also tend to have lower baseline levels of sIgA and less robust responses to stressors [58]. The potential use of sIgA as a non-invasive indicator of immunosuppression in the context of long-term emotional distress provides important new information on the relationship between psychological well-being and immunological competence. The predictive potential of salivary diagnostics in identifying persons at risk for stress-related health consequences might be enhanced by integrating it with other salivary indicators like cortisol and alpha-amylase [59].

Symptoms of anxiety and depression have been repeatedly linked to lower levels of salivary immunoglobulin A (sIgA). This is especially true in younger individuals, whose immune systems and emotional regulatory systems are still developing [60]. The ability to adjust to emotional difficulties is impaired and susceptibility to psychosocial stress is increased in those with lower sIgA concentrations [61].

Suppressed sIgA secretion is a hallmark of anxiety and depression in children, suggesting a type of psychologically driven immunosuppression in contexts like scholastic pressure, familial conflict, or early-life trauma [62]. Because of its inverse association, sIgA may be able to represent changes in neuroendocrine activity and impaired mucosal immunological defense, making it a promising integrative biomarker. So, it might be a great non-invasive way to find stress-related psychopathology and emotional dysregulation in kids and teens early on [63].

### 2.4. Chromogranin A (CgA)

The secretory vesicles of neuroendocrine cells, especially those located in the adrenal medulla and sympathetic nerve terminals, store and release chromogranin A (CgA) together with catecholamines, the most common of which are adrenaline and noradrenaline. As a critical component of the sympathetic–adrenal–medullary (SAM) system, CgA regulates neuroendocrine and immunological responses to stress and controls the release and storage of catecholamines [64]. Rapid secretion of CgA into circulation during acute psychological or physical stressor stimulation of the SAM axis makes it a potential non-invasive biomarker of sympathetic activity; it is detectable in saliva and other bodily fluids [65].

When the central nervous system processes a feeling of stress, the sympathetic nervous system increases its output, which in turn triggers the secretion of CgA [66]. Among these steps is the stimulation of brainstem and hypothalamic centers, which sets in motion a series of events that ultimately cause chromaffin cells to exocytose CgA [67].

**Figure 3:**
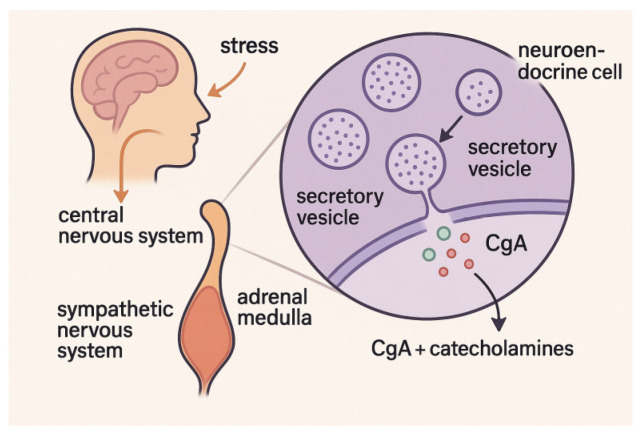
Chromogranin A (CgA) release pathway via the sympathetic–adrenal–medullary system during acute stress.

It provides an additional metric for SAM system activation when direct catecholamine assessment is not feasible because its release is highly correlated with that of catecholamines. The mechanism is illustrated in Figure 3 below.

Anxieties over public speaking, academic exams, performance anxiety, and other emotionally taxing situations have all been linked to elevated salivary CgA levels. As a result of its involvement in quick autonomic reactivity, its concentration often increases sharply in response to stressful situations and returns to normal levels soon after the stimulus has passed. Research has indicated that those who suffer from anxiety disorders or have heightened emotional sensitivity could display heightened sympathetic arousal through enhanced CgA responses [68]. Patients suffering from chronic stress or burnout may also have dysregulated CgA patterns, since the SAM system can remain activated for an extended period of time, which can cause changes to baseline levels or a reduction in reactivity [69].

There is growing interest in using CgA as a biomarker for emotional reactivity and stress vulnerability due to its high correlation with real-time physiological and emotional arousal. This could have applications in occupational stress monitoring, psychiatric assessment, and psychophysiological research. Its potential to improve stress profiling and shed light on subtle variations in stress response patterns may be realized by its combination with other salivary biomarkers like alpha-amylase and cortisol [70].

### 2.5. Interleukin-6 (IL-6)

An important regulator of immunological responses, acute-phase reactions, and inflammation is interleukin-6 (IL-6), a pleiotropic pro-inflammatory cytokine. Because of its role in the molecular mechanisms underpinning stress-related psychopathology, it is attracting more and more interest as a salivary biomarker [71]. Anxiety, sadness, and chronic stress are all mental health issues that are associated with elevated IL-6 saliva levels, which can be used as a marker of dysregulated immune-neuroendocrine function [72].

Activation of the sympathetic–adrenal–medullary (SAM) system and the hypothalamic–pituitary–adrenal (HPA) axis both induce the production of interleukin-6 (IL-6). When under stress, the immune system and epithelial tissues are influenced by circulating catecholamines and glucocorticoids, which mainly trigger the production of IL-6 [73].

Chronic exposure and reduced glucocorticoid receptor sensitivity, which is typical in major depressive disorder (MDD), might paradoxically boost IL-6 production, despite glucocorticoids’ conventionally inhibitory nature. As a feature of long-term mental health issues, this process reveals a transition to a pro-inflammatory state [74].

IL-6 contributes to the development of mood and anxiety disorders through several interrelated mechanisms. It promotes neuroinflammation by crossing the blood–brain barrier or being locally produced in the CNS, leading to glial activation [75]. Elevated IL-6 disrupts neurotransmitter systems, particularly serotonin, dopamine, and glutamate, affecting mood and cognition. It impairs HPA axis feedback, sustaining cortisol release and stress sensitivity [76]. Additionally, IL-6 activates indoleamine 2,3-dioxygenase (IDO), diverting tryptophan metabolism toward kynurenine rather than serotonin, which is linked to depressive symptoms, fatigue, and cognitive dysfunction [77].

Acute stressors, including public speaking or social evaluation tasks, raise IL-6 levels, which may be consistently measured in saliva. The peak of this rise usually happens 30 to 60 min after the stimulus. Individuals with heightened inflammatory tones may be identified by the continuously higher baseline salivary IL-6 levels linked with chronic psychological stress.

Salivary IL-6 levels in people with MDD and GAD are greater than in healthy controls, according to research. These levels may be correlated with the severity of symptoms. Supporting its significance in developmental stress evaluation, increased salivary IL-6 in pediatric populations has been associated with Adverse Childhood Experiences (ACEs), emotional neglect, and early-life trauma [78].

Despite its promising potential, interpretation of salivary IL-6 requires careful consideration of confounding factors, including local oral inflammation, circadian variability, and individual immune responsiveness. Nonetheless, when combined with other salivary biomarkers such as cortisol or alpha-amylase, IL-6 may enhance the predictive accuracy of psychophysiological models used to detect and monitor anxiety, depression, and stress-related disorders.

### 2.6. Tumor Necrosis Factor-Alpha (TNF-α)

One important pro-inflammatory cytokine that plays a role in immune system regulation, cell signaling, and apoptotic pathways is tumor necrosis factor-alpha (TNF-α) [79]. A key player in the inflammatory cascade, it is mostly released by monocytes and activated macrophages in reaction to stress, illness, or injury [80]. An encouraging salivary biomarker for emotional dysregulation, elevated TNF-α levels have been regularly seen in those with major depressive disorder (MDD), generalized anxiety disorder (GAD), and persistent psychosocial stress [81].

Psychological stress activates the HPA axis and the sympathetic nervous system, leading to increased production of TNF-α both centrally and peripherally. This cytokine acts through two primary receptors—TNFR1 and TNFR2—which modulate a wide range of cellular responses, including apoptosis, inflammation, and neuroplasticity [82].

Salivary TNF-α reflects mucosal and systemic inflammatory states and has been found to be elevated in individuals under chronic psychological distress. Studies show a positive correlation between salivary TNF-α and depressive symptom severity, particularly in individuals with somatic complaints or treatment-resistant depression [83].

TNF-α contributes to psychopathology through multiple mechanisms. It induces neuroinflammation by activating microglial cells and disrupting synaptic signaling. Elevated levels in the hippocampus impair neurogenesis and synaptic plasticity, affecting emotional and cognitive regulation [84]. TNF-α also contributes to serotonergic dysfunction by increasing serotonin transporter expression and activating indoleamine 2,3-dioxygenase (IDO), which diverts tryptophan from serotonin synthesis. Additionally, it promotes oxidative stress via enhanced ROS production, leading to neuronal damage in mood-related brain regions [85].

Its non-invasive detection makes it valuable for early identification of inflammatory dysregulation in at-risk individuals, especially when used in combination with other salivary biomarkers such as IL-6 and cortisol [86].

### 2.7. C-Reactive Protein (CRP)

In reaction to inflammation, especially when stimulated by interleukin-6 (IL-6), the liver synthesizes C-reactive protein (CRP), an acute-phase protein. Along with its roles in host defense, tissue regeneration, and immunological control, it is a clinically recognized indicator of systemic inflammation [87]. While C-reactive protein is often evaluated in blood, new research has shown that it may also be found in saliva. This provides a non-invasive way to evaluate inflammatory states linked to mood disorders and psychological stress [88].

The HPA axis is activated and pro-inflammatory cytokines like IL-6 and TNF-α are released when people experience psychological stress. This, in turn, triggers hepatocytes to manufacture CRP. Anxieties, major depressive disorder (MDD), and chronic stress are all associated with systemic low-grade inflammation, which is reflected in an elevated C-reactive protein (CRP) [89]. Autonomic imbalance and stress-induced glucocorticoid resistance may keep this inflammatory response going strong.

Although CRP does not cross the blood–brain barrier, elevated levels are linked to neuroinflammatory changes affecting brain function. High CRP is associated with altered connectivity in emotion-related networks (e.g., prefrontal cortex, amygdala), reduced serotonin and dopamine availability due to cytokine-driven enzymatic shifts, and symptoms like fatigue, cognitive impairment, and somatic complaints. Elevated CRP also predicts poor response to antidepressants and higher risk of treatment resistance [90].

One potential non-invasive method for evaluating systemic inflammation in mental health populations is salivary C-reactive protein. Salivary CRP concentrations are higher in those who report high levels of perceived stress or who exhibit clinical symptoms of anxiety and depression, according to studies. Anhedonia, psychomotor slowness, and sleep difficulties are among the behavioral symptoms that have been associated with high salivary CRP [87].

An important element of personalized medicine is finding reliable biomarkers for stress, anxiety, and depression, especially for predicting how well a patient will respond to therapy or how bad their prognosis would be. On the other hand, a lot of effort is required at the mechanical level as well. Mood and anxiety disorders, such as post-traumatic stress disorder (PTSD), are associated with elevated C-reactive protein levels, which in turn are associated with altered morphology and altered activation of the threat circuit [91].

### 2.8. Brain-Derived Neurotrophic Factor (BDNF)

Neurogenesis, synaptic plasticity, and neuronal survival are all aided by the neurotrophin brain-derived neurotrophic factor (BDNF). It is essential for the maturation of the brain, learning, memory, and emotional control [92]. BDNF has a significant role in the pathogenesis of stress-related diseases, major depressive disorder (MDD), and generalized anxiety disorder (GAD) [93]. While BDNF has long been evaluated in blood samples like serum or plasma, it has recently been found in saliva as well, making it a potential non-invasive biomarker for mental health issues associated with dysfunctions in neuroplasticity [94,95].

Areas of the brain that are important in cognition and emotion, such as the prefrontal cortex, hippocampus, and amygdala, are the primary sites of BDNF expression [96]. Glucocorticoids activate the HPA axis, which modulates its expression, and it is extremely sensitive to environmental stress. Decreased trophic support for neurons and impaired synaptic signaling are the results of downregulation of the BDNF gene expression brought on by chronic psychological stress and high cortisol levels [97].

There seems to be a two-way street between immunological activity and neuroplasticity, as inflammatory cytokines like IL-6 and TNF-α have a detrimental effect on BDNF production [98].

New research shows that BDNF may be found in saliva and that there is a modest correlation between salivary levels and central and serum concentrations. People who report high levels of perceived stress, depressive symptoms, and emotional tiredness often have lower amounts of BDNF in their saliva [99]. When blood samples are not an option, this non-invasive salivary test can help track neuroplasticity and therapy efficacy. One obstacle to broad clinical deployment is the lack of sufficient data and the technical heterogeneity in salivary BDNF measurement, which includes factors like stimulated vs. unstimulated saliva and test sensitivity.

### 2.9. Salivary MicroRNAs (miRNAs)

Typically ranging in length from 18 to 25 nucleotides, microRNAs (miRNAs) are short non-coding RNA molecules that control gene expression after transcription by attaching to certain messenger RNAs (mRNAs) and causing their destruction or translational suppression. They have an essential role in regulating the immune system, synaptic plasticity, and neurodevelopment, among other cellular processes. Several mental illnesses, such as anxiety, depression, and stress-related diseases, have been linked to the dysregulated expression of certain miRNAs [100].

The presence of extracellular vesicles (such as exosomes) in saliva shields miRNAs from enzymatic degradation and reflects molecular signals received from both the systemic and brain regions, making saliva a promising source for miRNA study [101].

Psychological stress, neuroinflammation, and dysregulated glucocorticoid signaling alter miRNA expression profiles that control genes related to neuroplasticity, HPA axis regulation, and monoamine signaling [102]. For instance, miR-124 modulates HPA activity and neurogenesis; miR-16 regulates serotonin transporter expression and responds to antidepressants; and miR-134 influences dendritic spine morphology and emotional adaptability. Shifts in salivary miRNA levels reflect both acute stress responses and long-term vulnerability to psychopathology [103].

Stable, non-invasive, and detectable by quantitative reverse transcription polymerase chain reaction (qRT-PCR) and next-generation sequencing, miRNAs in saliva are promising clinical targets [104]. Early identification, treatment monitoring, and personalized treatments might be supported by specific profiles that identify individuals with depression, PTSD, or excessive stress. Normalization variability, inter-individual variances, and lack of study standardization are some of the obstacles that restrict clinical translation [105] (Table 1).

### 2.10. S100 Proteins

The S100 protein family, composed of low-molecular-weight calcium-binding proteins, plays a central role in intracellular and extracellular regulatory activities, including cell proliferation, differentiation, and inflammation. Among them, S100B is particularly relevant to psychiatry due to its expression in astrocytes and its involvement in neuroplasticity and neuroinflammation [106]. Elevated serum S100B levels have been reported in individuals with major depressive disorder (MDD), bipolar disorder, and anxiety, often correlating with disease severity, glial dysfunction, and increased blood–brain barrier permeability [107].

Recent pilot studies have detected S100B in saliva, indicating its potential utility as a non-invasive biomarker, although its diagnostic specificity and salivary kinetics are not yet fully established [108]. The release of S100B into saliva may reflect neuroinflammatory signaling or peripheral glial responses to chronic stress and emotional dysregulation. [109].

Despite the currently limited body of research, salivary S100B represents a promising adjunct biomarker that may complement established salivary analytes such as cortisol, cytokines, or neurotrophic factors in reflecting the complex neuroinflammatory and glial-related processes associated with affective disorders. Its detection in saliva offers a unique window into astrocytic activity and central nervous system stress, potentially enhancing the diagnostic accuracy of salivary testing when used in combination with other physiological markers [110].

However, further investigation is critically needed to validate the clinical relevance of salivary S100B. This includes large-scale studies aimed at establishing baseline levels in healthy and clinical populations, exploring its temporal dynamics in response to stress or treatment, and determining cut-off values with adequate sensitivity and specificity for psychiatric screening.

In addition, efforts must be made to standardize sample collection, storage, and assay methodologies, as current variability in these procedures significantly limits the generalizability of findings. With these challenges addressed, salivary S100B could become an integral component of non-invasive, multibiomarker-based assessment tools in mental health care.

### 2.11. Additional Biomarkers of Potential Relevance

Although the current review has primarily focused on well-studied salivary biomarkers such as cortisol, alpha-amylase, cytokines, and neurotrophic factors, a number of other molecular candidates have emerged in recent years as potentially relevant to psychiatric conditions—particularly due to their involvement in neuroinflammation, neurovascular integrity, and cellular stress responses [111].

Among these, matrix metalloproteinases (MMPs)—notably MMP-2 and MMP-9—have attracted increasing interest in the field of neuropsychiatry. These zinc-dependent enzymes play a central role in extracellular matrix degradation, blood–brain barrier permeability, and regulation of neuroinflammatory cascades, all of which are pathophysiological processes implicated in depression, anxiety, and chronic stress [112]. Elevated levels of MMP-9 in serum and cerebrospinal fluid have been linked to structural and functional brain changes, such as hippocampal atrophy and synaptic remodeling, observed in patients with major depressive disorder or PTSD [113].

Recent studies have reported the presence of MMPs in saliva, although this evidence remains scarce and largely exploratory. The ability to detect MMPs in oral fluids may reflect their peripheral release during systemic or neurogenic inflammation and suggests a novel route for non-invasive monitoring of neuroinflammatory burden in mental health contexts [114].

One important limitation that must be acknowledged is the potential confounding influence of local oral inflammatory conditions, particularly periodontitis, on salivary biomarker levels. Periodontitis, as a chronic and prevalent oral disease, is associated with increased production of pro-inflammatory cytokines (such as IL-1β, IL-6, and TNF-α), as well as elevated oxidative stress markers and matrix metalloproteinases (e.g., MMP-8, MMP-9) [115].

These mediators may independently alter the salivary profile, thereby mimicking or amplifying the biomarker patterns typically associated with systemic stress, anxiety, or depression. Moreover, periodontitis can affect mucosal permeability and salivary composition, contributing to variability in cortisol, alpha-amylase, and immunoglobulin A levels [116].

Given the bidirectional link between poor mental health and oral hygiene neglect, it is essential that future studies systematically assess and control for periodontal status, either through exclusion criteria or statistical adjustment. Failing to account for such local oral factors may compromise the specificity and interpretability of salivary diagnostics in psychiatric settings.

Nevertheless, most of the studies included are heterogeneous in design, scale, and methodology, making it difficult to draw definitive conclusions. A synthesis of representative studies, including their design, sample size, investigated biomarkers, and limitations, is available in Table 2 below.

## 3. Future Perspectives

The increasing focus on salivary biomarkers as instruments for evaluating mental health is indicative of a larger trend toward non-invasive diagnostics and precision psychiatry. Several important avenues for further study and clinical integration are becoming apparent as more and more information becomes available.

The establishment of universally accepted procedures for the gathering, processing, and analysis of samples is an urgent requirement. The current state of clinical translation is hindered by methodological differences, which make it difficult to compare results across trials. To transition from data collected in experiments to diagnostic tools, it is necessary to establish consensus recommendations and established reference ranges that account for diurnal fluctuation, oral health status, age, sex, and lifestyle variables.

In the future, it may be possible to measure stress and emotional changes in real time in realistic settings thanks to technological advancements like point-of-care testing devices, wearable biosensors, and digital applications that combine salivary analysis with behavioral tracking. This paves the way for more flexible approaches to health care, shifting focus from reactive to proactive and adaptable measures.

Also, it is possible to increase diagnostic specificity by predicting treatment results and risk stratification using salivary biomarkers in conjunction with machine learning algorithms. In the long run, these resources may allow for more efficient and effective mental health care delivery through the development of individualized treatment programs.

Future research should focus on developing multimarker salivary diagnostic panels that integrate the complementary strengths of biomarkers such as cortisol, salivary alpha-amylase (sAA), and inflammatory or neurotrophic mediators. The rationale behind this approach lies in the multifactorial pathophysiology of stress-related disorders, where dysregulations in endocrine, autonomic, and immune pathways often coexist and interact.

By combining indicators from the hypothalamic–pituitary–adrenal (HPA) axis (e.g., cortisol), the autonomic nervous system (e.g., sAA), and the neuroimmune interface (e.g., cytokines, neurotrophins, S100B), researchers may capture a more holistic and biologically relevant profile of the individual stress response. This system-level integration could significantly enhance the diagnostic sensitivity and specificity for anxiety, depression, and chronic stress conditions, compared to isolated biomarker evaluation.

Moreover, such panels hold promise for the development of non-invasive, point-of-care screening tools, with potential applications in preventive mental health care, early diagnosis, and treatment monitoring. To achieve this goal, future studies should focus on standardizing collection protocols, validating biomarker stability in saliva, and establishing clinically meaningful thresholds through large-scale, longitudinal research across diverse populations.

Despite their potential, salivary biomarkers should not be seen in isolation. Collaboration among neuroscientists, data scientists, molecular biologists, and physicians is crucial for their potential therapeutic value. Thoughtful frameworks will also be required to address ethical concerns around psychological labelling, biomarker interpretation, and privacy when these techniques are used more often.

## 4. Conclusions

Salivary biomarkers serve as a significant, non-invasive method for identifying and tracking psychiatric problems, including anxiety, depression, and chronic stress. Their physiological significance—indicated by HPA axis activity, autonomic function, immunological response, and neuroplasticity—provides a comprehensive view of the biological foundations of mental health.

Cortisol and alpha-amylase are the primary indicators of acute stress response, whereas cytokines like IL-6 and TNF-α, in conjunction with CRP, signify chronic inflammation linked to prolonged emotional distress. Secretory IgA, BDNF, and salivary microRNAs offer more understanding of immunosuppression, synaptic dysfunction, and gene regulation, respectively.

Notwithstanding promising results, the practical use of salivary biomarkers necessitates the standardization of collection and analytical techniques, the management of confounding factors, and the integration with behavioral and clinical data.

Future advancements in biosensing technology and computer modelling may improve their prediction accuracy and diagnostic precision. The integration of molecular biology, psychoneuroendocrinology, and digital health technology underscores the promise of salivary biomarkers in enhancing personalized mental health treatment.

## Figures and Tables

**Table 1:** Comparative table of salivary biomarkers in anxiety, depression, and stress.

Biomarker	System Represented	Relevance in Mental Health	Detection Method	Limitations	Temporal Applicability	Saliva Type
Cortisol	HPA axis	Elevated in stress, depression; reflects HPA dysregulation	ELISA, immunoassay	High circadian variability, influenced by medications	Acute	Unstimulated
Alpha-amylase (sAA)	SAM system	Rapidly increases under acute stress; reflects sympathetic activity	Enzymatic assay	Affected by salivary flow rate, transient changes	Acute	Stimulated or resting (often both)
Secretory IgA (sIgA)	Mucosal immunity	Reduced in chronic stress and depression; indicates immune suppression	Immunoassay (ELISA)	Sensitive to oral health and local inflammation	Acute/Long-Term	Stimulated
Chromogranin A (CgA)	SAM system	Correlates with sympathetic arousal; rises during acute stress	Immunoassay	Requires timing; overlaps with other markers	Acute	Stimulated
Interleukin-6 (IL-6)	Immune–inflammatory	Elevated in chronic stress, anxiety, and depression; promotes inflammation	ELISA, multiplex cytokine panels	Low specificity, varies with comorbid conditions	Acute/Long-Term	Unstimulated
Tumor Necrosis Factor-alpha (TNF-α)	Immune–inflammatory	Linked to neuroinflammation and neurotransmitter imbalance in depression	ELISA, multiplex cytokine panels	Overlap with other inflammatory markers; systemic effects	Acute/Long-Term	Unstimulated
C-Reactive Protein (CRP)	Systemic inflammation	Correlates with treatment resistance and fatigue in depression	ELISA, high-sensitivity immunoassay	Limited saliva standardization; influenced by infections	Long-Term	Stimulated
Brain-Derived Neurotrophic Factor (BDNF)	Neuroplasticity	Reduced in MDD and anxiety; linked to synaptic and cognitive dysfunction	ELISA, immunoassay	Low salivary concentrations; assay variability	Long-Term	Unstimulated
microRNAs (miRNAs)	Gene regulation	Differential profiles associated with depression, PTSD, stress	qRT-PCR, sequencing	Lack of standardization, normalization challenges	Long-Term	Unstimulated (standardized protocols recommended)

**Table 2:** Summary of representative studies on salivary biomarkers in affective disorders.

First Author/Year	Study Design	Sample Size	Population	Biomarkers Investigated	Key Findings	Evidence Strength/Limitations
Chojnowska et al., 2021 [10]	Review	N/A	General population—multiple studies	Cortisol, sAA, CgA, sIgA	Salivary biomarkers reflect HPA/SAM axis activation and mucosal immunity in stress and affective disorders	Narrative synthesis; lacks unified methodology
Tammayan et al., 2021 [11]	Cross-sectional	n = 90 students	Dental students under exam stress	Cortisol, sAA, CgA	Elevated salivary cortisol and sAA correlated with subjective stress scores	Good sample; limited to academic context
Gholami et al., 2017 [12]	Clinical comparative	n = 60 (30 depressed, 30 controls)	Depressed vs. healthy adults	Salivary flow rate	Depressed patients had significantly reduced salivary flow rate	Small sample; focused only on xerostomia
Shimizu et al., 2024 [15]	Pilot crossover study	n = 18 psychiatric patients	Patients with depression/anxiety	Salivary biomarkers + physiological monitoring	Combined biometric and salivary analysis identified distinct stress response profiles	Pilot data; needs replication
Strahler et al., 2010 [53]	Experimental–age comparison	n = 60	Young, middle-aged, and older adults	Salivary alpha-amylase (sAA)	Stress-induced sAA reactivity varied by age; highest in younger adults	Age-diverse sample; limited to acute stressor design
Irshad et al., 2020 [61]	Observational (exam period)	n = unspecified	University students during exam stress	Cortisol, DHEA, salivary immunoglobulins, free light chains	Salivary biomarkers varied significantly during stress and were associated with infection symptoms	Multimodal biomarker approach; small sample size

## Abbreviations

The following abbreviations are used in this manuscript:

HPA	hypothalamic–pituitary–adrenal
SAM	sympathetic–adrenal–medullary
sAA	alpha-amylase
CgA	chromogranin A
sIgA	secretory immunoglobulin A
miRNAs	salivary microRNAs
IL-6	interleukin-6
CRP	C-reactive protein
TNF-α	tumor necrosis factor-alpha
BDNF	brain-derived neurotrophic factor
MDD	major depressive disorder
GAD	generalized anxiety disorder
WHO	World Health Organization
PVN	paraventricular nucleus
CAR	cortisol awakening response
CRF	corticotropin-releasing factor
ACTH	adrenocorticotropic hormone
SNS	sympathetic nervous system
CAMP	cyclic adenosine monophosphate
pIgR	polymeric immunoglobulin receptor
PKA	protein kinase A
PTSD	post-traumatic stress disorder
IDO	indoleamine 2,3-dioxygenase
ACEs	Adverse Childhood Experiences
qRT-PCR	quantitative reverse transcription polymerase chain reaction
